# Distinctive patterns and signals at major environmental events and collapse zone boundaries

**DOI:** 10.1007/s10661-021-09463-7

**Published:** 2021-09-29

**Authors:** Melinda Pálinkás, Levente Hufnagel

**Affiliations:** 1 Doctoral School of Environmental Sciences, Hungarian University of Agriculture and Life Sciences, Gödöllő, Hungary; 2 Research Institute of Multidisciplinary Ecotheology, John Wesley Theological College, Budapest, Hungary

**Keywords:** Warning signal, Collapse indicator, Climate change, Extreme event, Mass extinction, Dominant species

## Abstract

We studied the patterns of pre-collapse communities, the small-scale and the large-scale signals of collapses, and the environmental events before the collapses using four paleoecological and one modern data series. We applied and evaluated eight indicators in our analysis: the relative abundance of species, hierarchical cluster analysis, principal component analysis, total abundance, species richness, standard deviation (without a rolling window), first-order autoregression, and the relative abundance of the dominant species. We investigated the signals at the probable collapse triggering unusual environmental events and at the collapse zone boundaries, respectively. We also distinguished between pulse and step environmental events to see what signals the indicators give at these two different types of events. Our results show that first-order autoregression is not a good environmental event indicator, but it can forecast or indicate the collapse zones in climate change. The rest of the indicators are more sensitive to the pulse events than to the step events. Step events during climate change might have an essential role in initiating collapses. These events probably push the communities with low resilience beyond a critical threshold, so it is crucial to detect them. Before collapses, the total abundance and the species richness increase, the relative abundance of the species decreases. The hierarchical cluster analysis and the relative abundance of species together designate the collapse zone boundaries. We suggest that small-scale signals should be involved in analyses because they are often earlier than large-scale signals.

## Introduction

The current climate change and the ongoing sixth mass extinction (Barnosky et al., 2011) highlight the importance of research on community collapses (Henderson, 2007; Lever et al., 2014; Dakos & Bascompte, 2014; Hufnagel et al., 2020). The anthropogenic global warming and the direct human effects are changing the habitats so quickly that many species cannot adapt to it and are drifting to extinction (Di Nitto et al., 2014; Pershing et al., 2016). It is essential to recognize the distinct pattern and the warning signals of the decline and the collapse of communities in a longer perspective.

We believe that communities show changes in their patterns long before collapses. These changes result in decreased resilience and a higher tendency for a collapse. Hence, unusual environmental events can quickly push the communities past a critical threshold leading to a collapse (Matusick et al., 2013; Valenzuela et al., 2019). Unusual or extreme environmental events are sudden and of great magnitude. They can be pulse events or step events. We can describe pulse events as a sudden and significant change of system parameters for a short period, for example, bolide events, volcanic eruptions, sudden, temporary cooling or warming, fires. During step events, the system parameters also change suddenly, but these changes last much longer. Step events are typically related to climate changes when the temperature rises or drops considerably and stays high or low for an extended time. Unusual environmental events are stochastic, and it is difficult or impossible to predetermine their time windows. These events probably have an essential role in the initiation of community collapses. In this study, we involved both pulse events and step events. Pulse events are Cretaceous-Paleogene bolide event, Paleocene-Eocene presumed pulse event before Paleocene-Eocene Thermal Maximum, Early Miocene transient glaciation, and Early Miocene presumed temporary warming. Step events are Paleocene-Eocene Thermal Maximum, Arctic warming events during the 1920s–1940s, presumed EM2, EM1.

Some species have a more significant effect on the community structure than others do. Dominant species, the most abundant species of the communities, are essential in maintaining the communities (Power et al., 1996). It is important to note that no uniform definition for the dominant species exists in the literature. Some are based simply on their high abundance (e.g., Odum & Barrett, 2005; Rabinowitz, 1981; Ricklefs et al., 1999); others also emphasize their high impact within the community (e.g., Clements, 1907; Dayton, 1971; Power et al., 1996; Whittaker, 1965). They probably have a fundamental role in maintaining ecosystem functions and diversity. The removal of dominant species reduces ecosystem functions and significantly affects diversity (Avolio et al., 2019). According to Grime (1998), a species affects an ecosystem function in proportion to its relative abundance in a community. Based on this concept, we assume that the most abundant species is the most influential species among abundant species in a community.

In this study, we apply the phrase of dominant species only to the most abundant species of the communities. The decline of a dominant species causes changes in the occurrence and distribution of other species and modifies the processes and structures of communities. Their abundance probably also provides a delay in collapses after the environmental events. The most abundant species are likely to be sensitive to environmental changes because they gain their abundance by adapting the best to their environment. Arnoldi et al. (2019) suggest that common species generate the response of a community to environmental disturbances. Significant, negative environmental changes (unusual, extreme events) significantly affect the abundance of the dominant species. According to Parmesan et al. (2000), extreme events provoke a wide range of ecological responses scaling from the gene to the ecosystem, and they are essential components of population extinctions. We presume that collapse triggering environmental events cause a sharp drop in the abundance of the dominant species close to the event. These events, especially pulse events, may push the abundance of the dominant species below a critical level.

The decline of the dominant species because of an unfavorable environment can benefit the sub-dominant species, opportunistic species, and disaster species. They appear before the collapse, which can cause an increase in the species richness. This phenomenon can be observed under recent climate change, as well. Tropical and boreal species expand their ranges and invade temperate and polar communities, respectively (Parmesan et al., 2000; Scheffers et al., 2016). Lowland species are moving to higher elevations (Comte & Grenouillet, 2013; Freeman & Freeman, 2014; Wolf et al., 2016). We suppose that a sudden increase in species richness is often related to an unusual environmental event.

We involved critical slowing down indicators in our analysis. Researchers use them as large-scale signal indicators. “Critical slowing down” (CSD) is a phenomenon of dynamical systems near critical transitions. At critical thresholds or tipping points (bifurcation), the complex dynamical systems shift abruptly from one state to another, called critical transition. Critical transitions or abrupt shifts can be observed in complex systems from medicine (Trefois et al., 2015) through finance (Gatfaoui & de Peretti, 2019; Lenton et al., 2008) to the climate system (Dakos et al. 2008; Dakos et al., 2015). In our case, this process is the community collapse (mass extinction, community shift). Once the system gets close to the critical threshold or tipping point, it slows down, taking a longer time to recover from minor disturbances. The cause of this slowness in response is the low level of resilience. Commonly used CSD indicators are standard deviation, variance, lag-1 autocorrelation, and skewness. According to Dakos et al. (2015), CSD causes a rise in variance and temporal correlation. Flickering may be a reason for increasing variance (Dakos et al., 2013; Wang et al., 2012).

In the past few years, authors have become more critical of CSD indicators. CSD indicators do not always increase before critical transitions (Dakos et al., 2012b; Wang et al., 2012). Guttal et al. (2013) suggest that variance is not a reliable CSD indicator because it decreases or increases before regime shifts. Sometimes, regime shifts have no tipping points, and in this case, there is no CSD at regime shifts (Dakos et al., 2012a, b). Many range shifts are long and smooth after the tipping point, and it is difficult to detect them, which delays management actions (Hughes et al., 2013). Burthe et al. (2016) tested CSD indicators on 125 long-term abundance time-series of 55 taxa “across multiple trophic levels in marine and freshwater ecosystems,” and they concluded that they are not effective in predicting nonlinear changes. Biggs et al. (2009) and Spanbauer et al. (2014) propose that a significant increase in collapse indicators happens at the onset of collapses, which is too late for intervention.

This study investigates the pre-collapse pattern of communities, and the signals of major environmental events and community collapses, respectively. We want to focus on the early warning signals of collapses and the signs of the collapse triggering major environmental events. Authors tend to use only a few indicators to analyze community collapses. However, various aspects of collapses can thereby remain hidden. They mainly research large-scale signals of collapses using CSD indicators, and they neglect small-scale signals. Other research fields use small-scale signal detection more frequently. Civil engineers use outlier detection in the structural damage diagnosis of the built infrastructure (Kim et al., 2013). They also apply transient signals for the same purpose (Opoka et al., 2013). In financial market analysis, they use outliers as early warning signals of a financial crisis (Laubsch, 2015).

We presume that we can learn more about the general pattern of collapses if we analyze collapses of different causes, different data types at different scales. We examined five data series of collapses (mass extinctions and a modern community shift) with various statistical indicators. Besides using community-level indicators (relative abundance of species, hierarchical cluster analysis [HCA], principal component analysis [PCA], total abundance, species richness, first-order autoregression [AR1], and standard deviation), we also introduced a new indicator at the species level: the relative abundance of the dominant species (the most abundant species in the community). Dominant species quickly respond to environmental events and are likely to be an essential biological contributor to community collapses. The dominant species has not been studied as a collapse indicator yet.

As unusual environmental events are also an interest of this study, we also examined whether the indicators are sensitive to pulse events, step events, collapse zone boundaries, and what signals they give.

### Glossary

We introduce/reintroduce some phrases for community collapses in this study in our understanding.

*Community collapse* – The collapse of the dominant species, the rise of a new dominant species, and the complete structural changes in the community indicate community collapse. We initiate using the “community collapse” phrase for community shifts and extinctions based on common features.

*Pre-collapse community* – Pre-collapse communities cover a period starting before the environmental event and ending at the collapse boundary.

*Collapse community* – Collapse communities follow pre-collapse communities, and they occupy the collapse zones where the pre-collapse dominant species are no longer the most abundant species.

*Dominant species* – Dominant species is the most abundant species of a community in this study. Its decline is an important biotic sign of a forthcoming collapse. We use pre-collapse dominant and collapse dominant species phrases in the study.

*Collapse zone vs. collapse point* – We used collapse zones instead of the points of the collapse (shift or extinction) because we did not intend to include collapse zone signals in the evaluation of indicators.

*Collapse zone* – We used the HCA clusters, the PCA, and the relative abundance of the dominant species to detect the collapse communities and the collapse zones (a period of collapse).

*Small-scale signals* – We use this phrase for statistically significant, temporary signals (peaks or troughs). Small-scale signals are values outside the 95% confidence interval.

*Large-scale signals* – Large-scale collapse signals are large-scale trend changes (change of the general, long-term direction) or slope changes (sudden change of steepness). The 95% confidence band usually shows the large-scale trends.

*Abbreviations of periods, epochs, and events:*

- KPG = Cretaceous-Paleogene
- PE = Paleocene-Eocene
- PETM = Paleocene-Eocene Thermal Maximum
- EM1 = Early Miocene 1
- EM2 = Early Miocene 2

## Materials and methods

### Data

We used open paleoecological data series available on the *PANGAEA—Data Publisher for Earth & Environmental Science* open access data library (https://pangaea.de/) and current paleolimnological data obtained from other authors (Lehnherr et al., 2018). The five studied data series range from the Cretaceous-Paleogene impact event to a recent shift, including different faunal records at different time scales. These data series are supplements to peer-reviewed articles, including the change points of time series (collapses), allowing us to check the accuracy of our results. All of them show abrupt, sudden changes in the community structures.

**Fig. 1:**
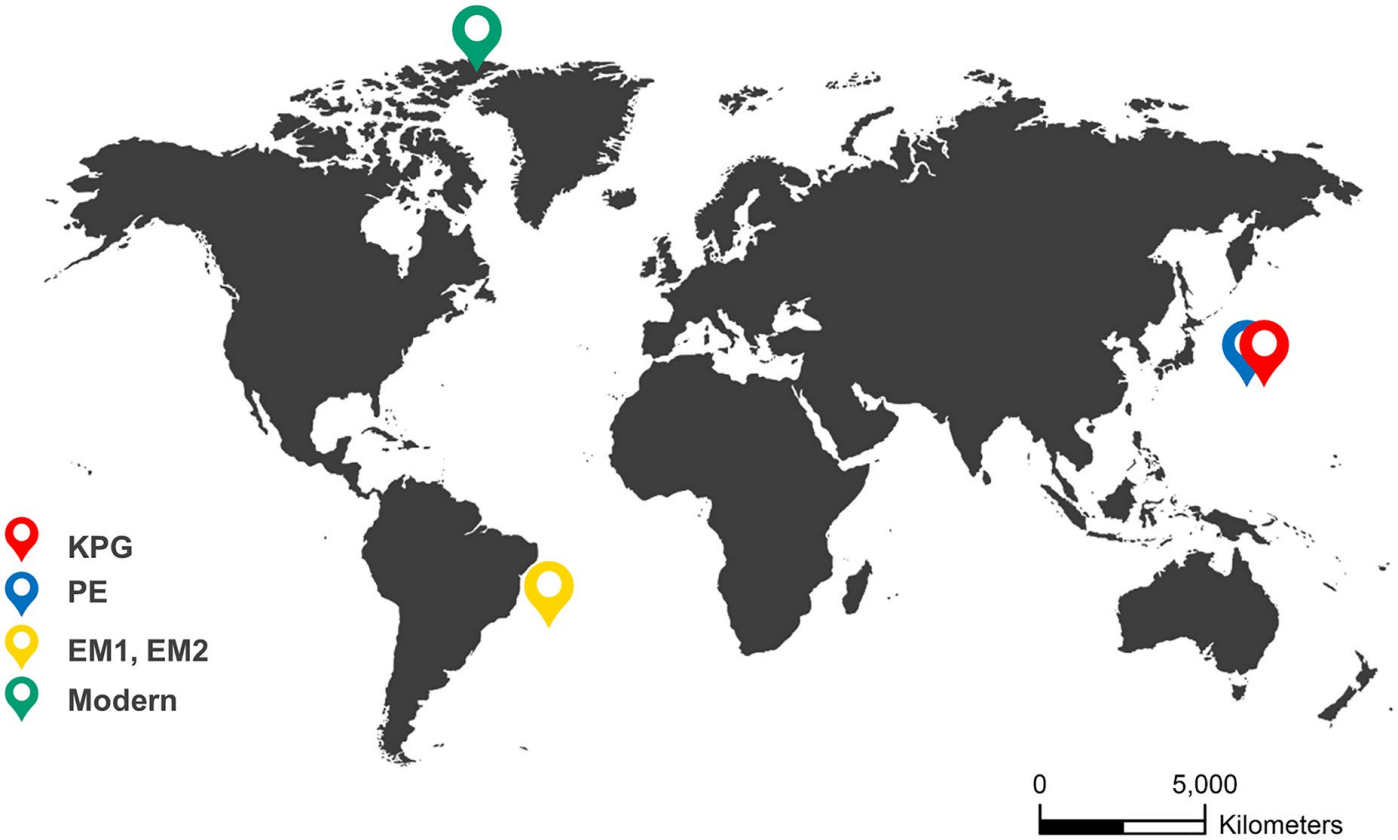
Locations of paleoecological and modern records. KPG = Cretaceous-Paleogene record of deep-sea benthic foraminifera. Shatsky Rise, northwest Pacific Ocean. Source: Alegret and Thomas (). PE = Paleocene-Eocene record of deep-sea benthic foraminifera. Shatsky Rise, northwest Pacific Ocean. Source: Takeda and Kaiho (2007b). EM1, EM2 = Early Miocene record of deep-sea nannofossils. South Atlantic Ocean. Source: Plancq et al. (2013b). Modern = modern record of diatoms. Lake Hazen, northern Ellesmere Island, Canada. Source: Köck et al. (2012) and Lehnherr et al. (2018). Source of world map template: www.pptbackgroundstemplates.com

**Fig. 2:**
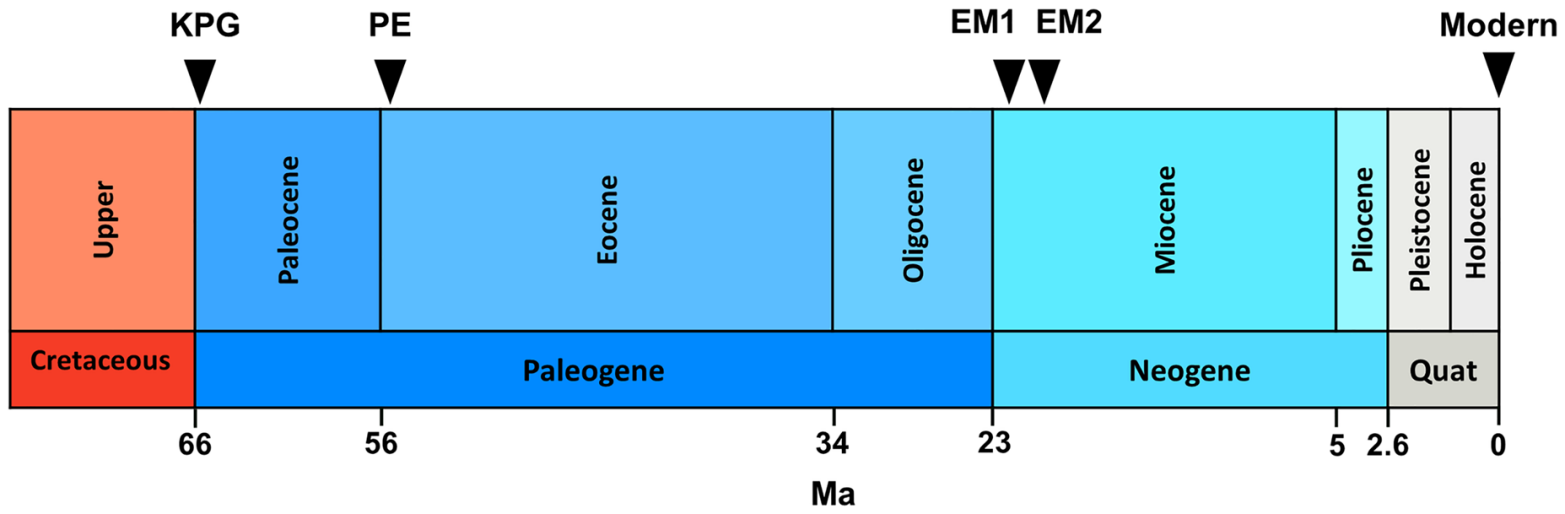
Timeline of environmental events and collapses. KPG = extinction of benthic foraminiferal species at the Cretaceous-Paleogene boundary (Alegret & Thomas, 2009a, b); probable impact event (65.7 Ma). PE = extinction of benthic foraminiferal species during the Paleocene-Eocene Thermal Maximum (Takeda & Kaiho, 2007b); probable warming event before PETM (55.08 Ma). EM1 = Early Miocene nannofossil extinction (Plancq et al., 2013a); probable glaciation event (21.3 Ma). EM2 = Early Miocene nannofossil extinction (Plancq et al., 2013a); probable warming event (20.2 Ma). Modern = recent diatom shift (1995–1997) (Lehnherr et al., 2018); probable warming events (1924; 1939). Ma = million years ago

The high-resolution Cretaceous-Paleogene record of deep-sea benthic foraminifera derives from depths between 1500 and 2000 m at the Northwest Pacific Ocean Drilling Program (ODP) Hole 198–1210A (Shatsky Rise, 32.2235°N, 158.2593°E; Fig. 1) (Alegret & Thomas, 2009a, b). Shatsky Rise is a large oceanic plateau 1500 km east of Japan. The sedimentological record shows a transient assemblage change of benthic foraminifera at the Cretaceous-Paleogene (K/Pg) boundary (between 219.9–219.8 mbsf ~ 65.489 Ma before present). It is probably a result of an impact event (Alegret & Thomas, 2009a, b). They collected the percent relative abundances of 101 species between 215.00 and 223.30 mbsf (meters below seafloor) corresponding with age between 64.70 and 66.14 Ma and consisting of 26 time steps (Fig. 2).

The Paleocene-Eocene sediments derive from ODP Hole 1212B (Shatsky Rise, 32.4485°N, 157.7117°E, water depth 2681 m; Fig. 1) (Takeda & Kaiho, 2007b). The high-resolution analysis revealed a major benthic foraminiferal extinction event during the Paleocene-Eocene Thermal Maximum (PETM) (Takeda & Kaiho, 2007a). The PETM interval at ODP Hole 1212B was between 55.0 Ma and 54.9 Ma (79.925–79.700 mbsf) (Takeda & Kaiho, 2007b). The relative abundances of 27 species come from a depth between 79.705and 80.005 mbsf covering 54.90–55.21 Ma with 31 time steps (Fig. 2).

The late Oligocene-early Miocene deep-sea nannofossil data come from the South Atlantic Deep Sea Drilling Program (DSDP) Site 516 (30.2763°S, 35.2852°W, water depth 1313 m; Fig. 1) situated about 1000 km away from the Brazilian coasts in the South Atlantic Ocean (Plancq et al., 2013a). The relatively warm climate of Early Miocene was interrupted with transient Antarctic glaciation events (Mi-1a and Mi-1aa events at about 21.3 Ma [approximately 170–180 mbsf] and 20.2 Ma [approximately 150–160 mbsf], respectively), which may be shown by the decline of *Cyclicargolithus floridanus*, the dominant species, in the record (Plancq et al., 2013b). They sampled the nannofossil assemblage between 104.46 and 239.05 mbsf, covering the Oligocene–Miocene boundary and the early Miocene (Fig. 2). It includes the relative abundances of 13 species with 33 time steps. We used two periods of the data series for analysis between 161.48–203.2 mbsf (EM1) and 109.78–157.45 mbsf (EM2), respectively. Both of them belong to the Early Miocene (EM) sub-epoch. EM1 probably includes a glaciation event, and EM2 involves a warming event.

The modern data series originates from a paleolimnological record of diatoms. The paleolimnological record of diatoms comes from the microfossil analysis of three sediment cores collected from Lake Hazen in 2013 (northern Ellesmere Island, Canada, near the deepest point of the lake [81.816806°N, 70.700222°W; Fig. 1], water depth 260 m) (Köck et al., 2012). Lake Hazen is the largest lake north of the Arctic Circle, situated in Quttinirpaaq National Park. The recent climate warming resulted in an ecological shift in the lake. The algal (diatom) primary producers shifted from shoreline benthic to open-water planktonic species probably because of the earlier onset of ice melting after winters and the longer growing seasons with a larger ice-free area between 1995 and 1997 (Lehnherr et al., 2018). The warming of the Lake Hazen watershed started more intensively around the turn of the twentieth century. Between 2000 and 2012, the mean summer land surface temperatures of ice-covered regions increased by 2.6 °C increase (Lehnherr et al., 2018). The relative abundances of 63 species refer to 1894–2014 (top 10–20 cm of the cores) with 18 time steps (Fig. 2).

### Methods

After organizing the data, we identified the pre-collapse, the collapse, and the recovery communities. Then, we selected the pre-collapse communities, including the environmental events and the boundaries between the pre-collapse and the collapse communities, to analyze the pre-collapse patterns and the signals of the environmental events and the collapse zones. To do so, we applied both univariate and multivariate indicators at the community level and the level of the dominant species. Finally, we evaluated the signals and the indicators.

#### Community-level indicators

We applied the relative abundance of the species (%), HCA (hierarchical cluster analysis), and PCA (principal component analysis) to define the communities and the unusual environmental events. We used multidimensional methods, temporal clustering, and principal component analysis (PCA), for dimension reduction in Past v 2.17 (Hammer et al., 2001).

We applied time-series clustering to detect communities and also unusual environmental events. In our hierarchical cluster analyses (HCA), we used an unweighted pair-group average (UPGMA) algorithm, for which we computed the distance matrix using Euclidean distance. We preferred constrained clustering to join the adjacent rows of time series during the agglomerative clustering. The products of our cluster analysis are dendrograms showing the clustered data points which represent the communities (before collapses, during collapses, after collapses) in the time series. We presume that the dendrograms also represent environmental pulse events as outliers. We also assume that environmental step events might give different signs.

We used principal component analysis (PCA) to explore the similarities between the clusters (communities). PCA shows how the communities are related to each other, and they provide information about the environmental events (climate change, unusual environmental events). We did the PCA by eigenvalue decomposition of a data covariance matrix. The convex hulls in the PCA scatterplots represent the communities.

The relative abundance of species (%) shows the unusual environmental events as a peak of the environmental indicator species and as a sharp drop of the pre-collapse dominant species at the same time. Identifying the dominant species of clusters/sub-clusters helps to define communities.

We used the indicators below to explore the pre-collapse pattern and the signals of the environmental events and the collapse zones.

*Total abundance* refers to the number of individuals in a community.

*Species richness* is the number of species in a community.

*Standard deviation* and *first-order autoregression* are the indicators of critical slowing down (e.g., Drake & Griffen, 2010; Scheffer, 2010). The standard deviation shows the variance in the data series compared to the average of the data series. We calculated the standard deviation in Excel (without applying a rolling window). We generated the AR(1) indicator by an autoregressive process of order 1. The process is a linear regression of the value of time *t* against that of time *t* − 1. We fitted the AR(1) model on the data with a 20-period rolling window using the earlywarnings R package (Dakos et al., 2012a).

#### Species-level indicator

We presume that the dominant species (the most abundant species) effectively detect the warning signs of collapses because the dominant species is probably sensitive to collapse triggering environmental events. It is one of the most influential species in a community (see “Introduction” section). We introduce the *relative abundance of the dominant species* as a possible environmental event indicator and perhaps a collapse indicator.

#### Statistical significance of collapse warning signals

We interpolated the data using *impuTS* R package so that we can do further analyses. We identified statistically significant warning signals of the indicators with a uniform method. We calculated the 95% pointwise confidence band for the indicators with *stats* R package. First, we fitted a polynomial curve to the data by loess R function (smoothing span = 1). We used the predict function for predictions from a loess fit with standard errors. Then, we calculated the upper and the lower boundaries of the confidence band with the following formula: pred$fit ± qt(0.95,pred$df)*pred$se, where pred = the prediction from loess fit, fit = the predicted values, df = an estimate of the effective degrees of freedom, and se = an estimated standard error for each predicted value. The fitted curve shows the general trends and the large-scale signs. Significant deviations from the confidence band are short-scale signs.

We applied Excel 2016, R 3.6.2, and ArcMap 10.4 for data visualization. We used R software (R Core Team, 2019) to create the figure of the statistical indicators (Fig. 10). We applied *ggplot2* and *patchwork* packages for the visualization of the statistical indicators. We georeferenced the world map of Fig. 1 in ArcMap 10.4.

## Results

### Identifying communities and boundaries

We applied the HCA and the relative abundance of species to identify the clusters and their boundaries. The clusters/sub-clusters represent the communities.

#### KPG

**Fig. 3:**
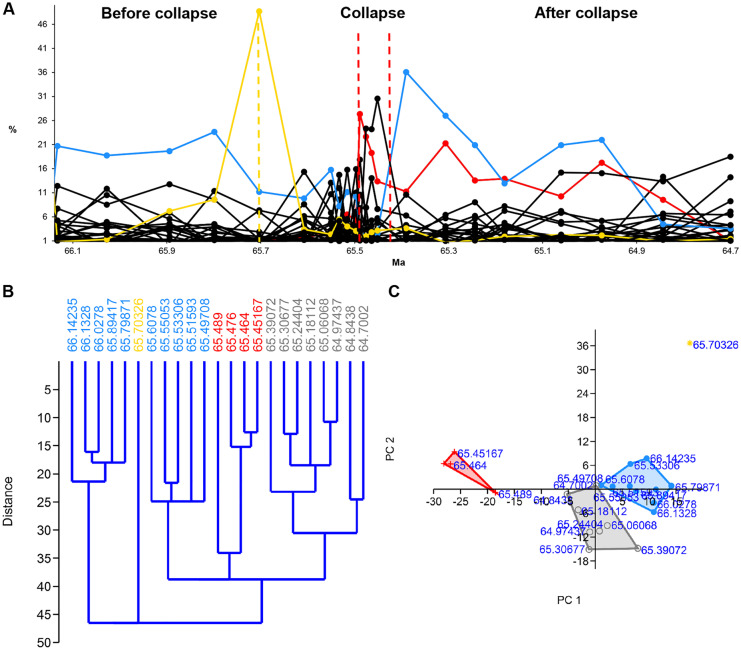
Relative abundance, hierarchical cluster analysis (HCA), and principal component analysis (PCA) of the benthic foraminiferal species across the K/Pg transition (KPG) (Northwest Pacific ODP). **A** The relative abundance of benthic foraminiferal species plot. Blue solid line = pre-collapse dominant species (*Nuttallinella ripleyensis*). Red solid line = collapse dominant species (*Bulimina kugleri).* Yellow solid line = environmental indicator (*Pyraminida rudita*). Yellow dashed vertical line = environmental pulse event (bolide event). Red dashed vertical line = collapse zone boundary. **B** HCA plot and **C** PCA plot. Blue = pre-collapse community, red = collapse community, grey = recovery community, yellow = environmental event (bolide event). Source of data: Alegret and Thomas ()

In the KPG data series, the pre-collapse community is in the interval between 66.14235 and 65.49708 Ma. This period includes two clusters between 66.14235–65.79871 Ma (cluster 1) and 65.6078–65.49708 Ma (cluster 2), respectively, as well as the bolide event between them 65.70326 Ma. The figure of the “Relative abundance of the benthic foraminiferal species across the K/Pg transition” and the HCA (Fig. 3A, B), cluster 2 gives the expression of a collapse zone. However, clusters 1 and 2 have the same dominant species, namely, *Nuttallinella ripleyensis* (Alegret & Thomas, 2009a, b). Hence, we suppose that cluster 2 belongs to the pre-collapse community. The collapse zone is cluster 3, in which the dominant species is *Bulimina kugleri* (Alegret & Thomas, 2009a, b)*.*

#### PE

**Fig. 4:**
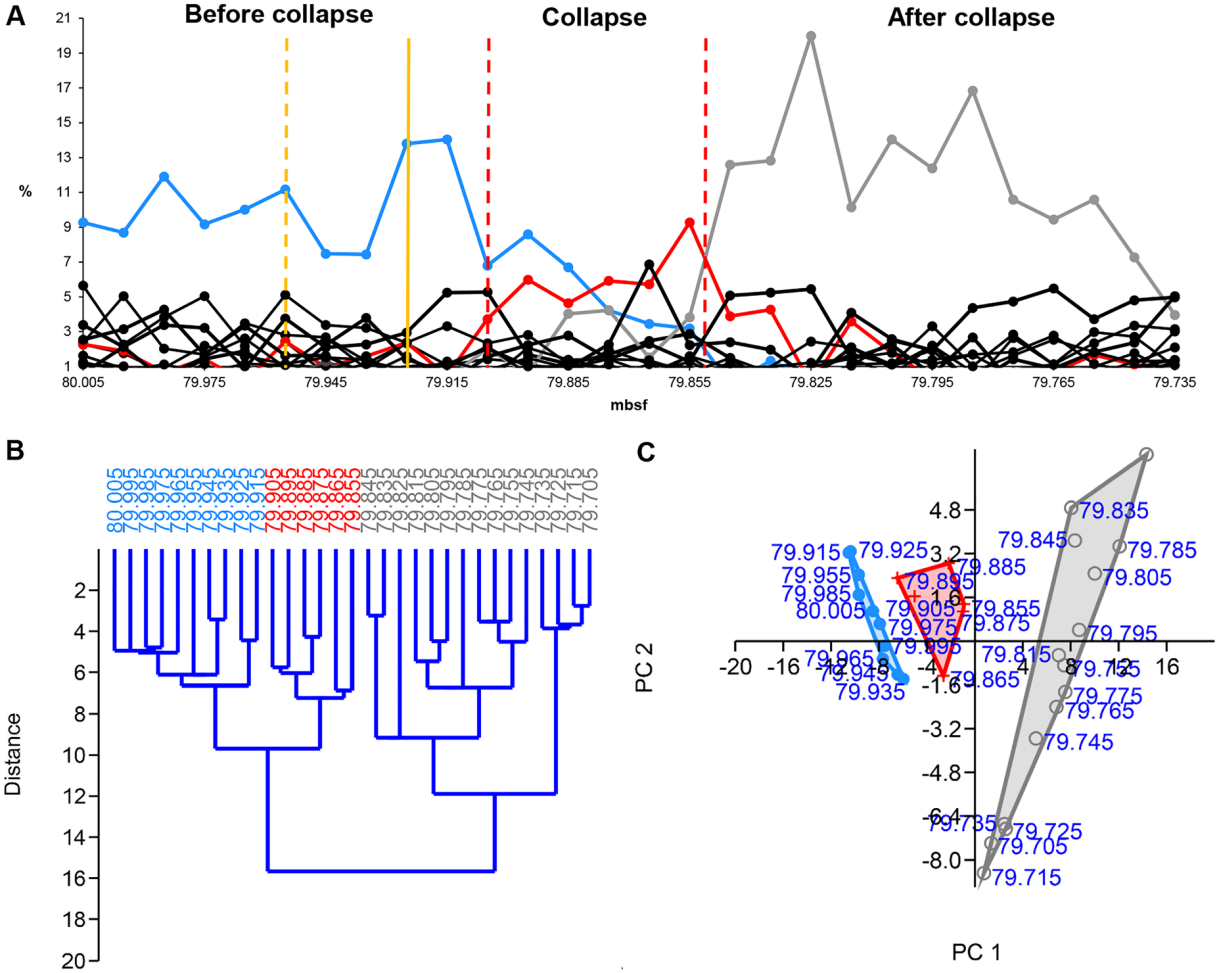
Relative abundance, hierarchical cluster analysis (HCA), and principal component analysis (PCA) of Paleocene-Eocene benthic foraminifera (Central Pacific, ODP Hole 1212B). **A** The relative abundance of benthic foraminiferal species plot. Blue solid line = pre-collapse dominant species (*Siphogenerinoides brevispinosa*). Red solid line = collapse dominant species (*Bolivina gracilis*). Grey solid line = recovery dominant species (*Quadrimorphina profunda*). Yellow dashed vertical line = presumed environmental pulse event. Yellow solid vertical line = environmental step event (PETM). Red dashed vertical line = collapse zone boundary. **B** HCA plot and **C** PCA plot. Blue = pre-collapse community, red = collapse community, grey = recovery community. Source of data: Takeda and Kaiho (2007b)

The HCA clearly indicates the pre-collapse community (80.005–79.915 mbsf) and the collapse community (79.905–79.855 mbsf) (Fig. 4B). The dominant pre-collapse species is *Siphogenerinoides brevispinosa* (Takeda & Kaiho, 2007b); the dominant collapse species is *Bolivina gracilis* (Takeda & Kaiho, 2007b).

#### EM1 and EM2

**Fig. 5:**
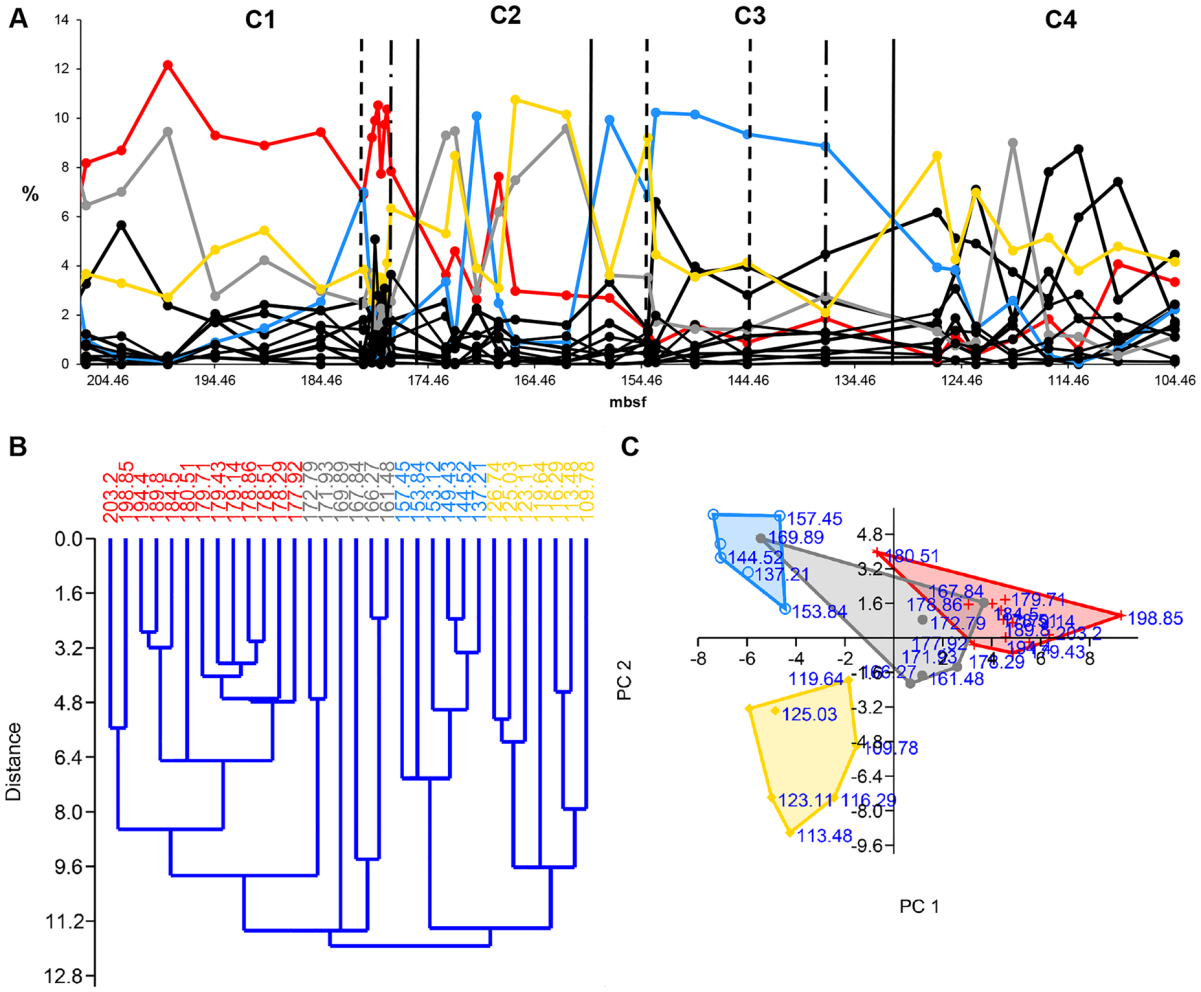
Relative abundance, hierarchical cluster analysis (HCA), and principal component analysis (PCA) of late Oligocene–Early Miocene nannofossils (South Atlantic DSDP Site 516). **A** The relative abundance of nannofossils. C1 = community 1 (EM1 pre-collapse community), C2 = community 2 (EM1 collapse community), C3 = community 3 (EM2 pre-collapse community), C4 = community 4 (EM2 collapse community). Red solid line = dominant species 1 (*Cyclicargolithus floridanus*). Grey solid line = dominant species 2 (*Dictyococcites* spp.). Blue solid line = dominant species 3 (*Dictyococcites antarcticus*). Yellow solid line = dominant species 4 (*Coccolithus* spp.). Black solid vertical line = community boundary, black dashed vertical line = presumed environmental pulse event, black dotdash vertical line = presumed environmental step event and/or community threshold. **B** HCA plot and **C** PCA plot. Red = community 1, grey = community 2, blue = community 3, yellow = community 4. Source of data: Plancq et al. (2013b)

**Fig. 6:**
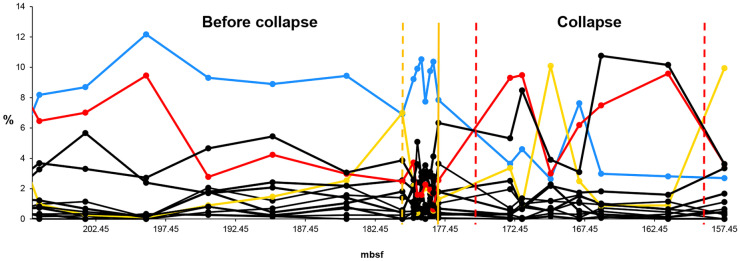
Relative abundance of Early Miocene nannofossils (EM1) (South Atlantic DSDP Site 516). Blue solid line = pre-collapse dominant species (*Cyclicargolithus floridanus*). Red solid line = collapse dominant species (*Dictyococcites* spp.). Yellow solid line = environmental indicator (*Dictyococcites antarcticus*). Yellow dashed vertical line = presumed environmental pulse event (temporary glaciation). Yellow solid line = presumed environmental step event and/or community threshold. Red dashed vertical line = collapse zone boundary. Source of data: Plancq et al. (2013b)

**Fig. 7:**
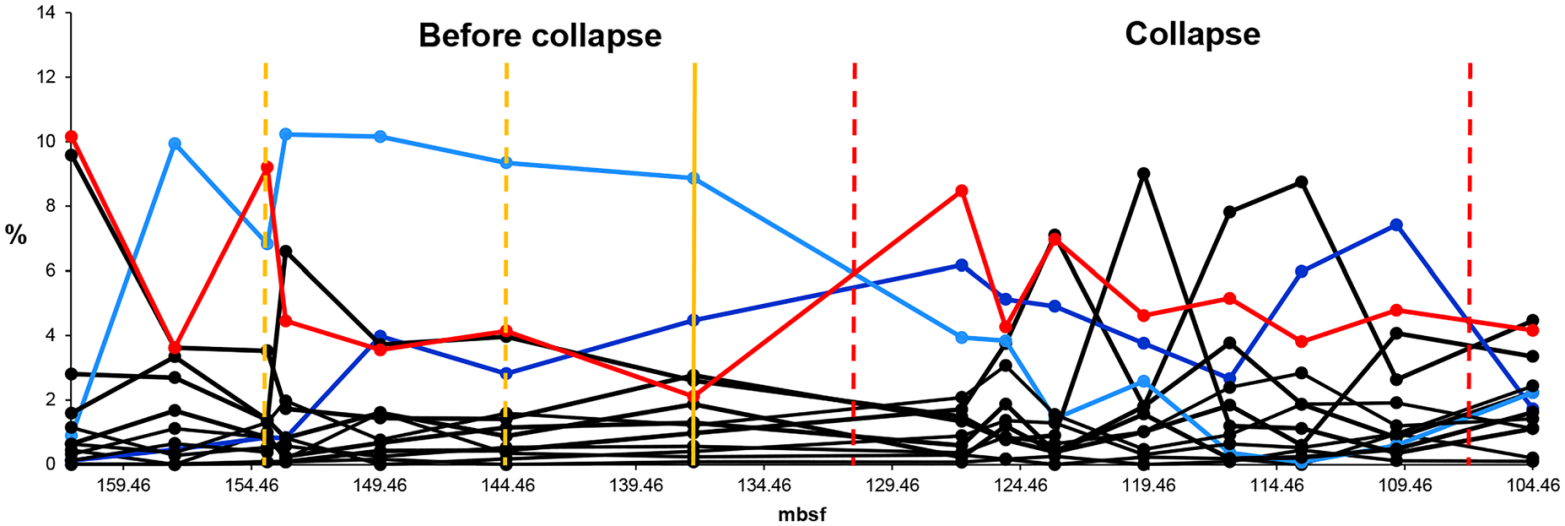
Relative abundance of Early Miocene nannofossils (EM2) (South Atlantic DSDP Site 516). Blue solid line = pre-collapse dominant species (*Dictyococcites antarcticus*). Red solid line = collapse dominant species (*Coccolithus* spp.). Yellow dashed vertical line = presumed environmental pulse event (temporary warming). Yellow solid vertical line = presumed environmental step event and/or community threshold. Red dashed vertical line = collapse zone boundary. Source of data: Plancq et al. (2013b)

The HCA of the original Late Oligocene-early Miocene data series shows four communities (community 1–4) (Fig. 5B). EM1 data series includes the collapse of community 1 (pre-collapse community) (Fig. 6). The HCA shows community 1 as cluster 1 between 203.2 and 171.93 mbsf. However, the relative abundance of species suggests that the boundary between community 1 and community 2 is earlier, between 177.92 and 172.79 mbsf. After 177.92 mbsf, *Dictyococcites* spp. (Plancq et al., 2013a), the dominant species of community 2 takes over the dominance from *Cyclicargolithus floridanus* (Plancq et al., 2013a), the dominant species of community 1. Hence, we decided to modify the boundary between community 1 and community 2 suggested by the HCA. EM2 involves the collapse of community 3 (pre-collapse community) (Fig. 7). The HCA shows community 3 as cluster 3 between 157.45 and 137.21 mbsf. The pre-collapse dominant species of EM2 is *Dictyococcites antarcticus* (Plancq et al., 2013a). The collapse dominant species are *Coccolithus* spp. (Plancq et al., 2013a).

#### Modern

**Fig. 8:**
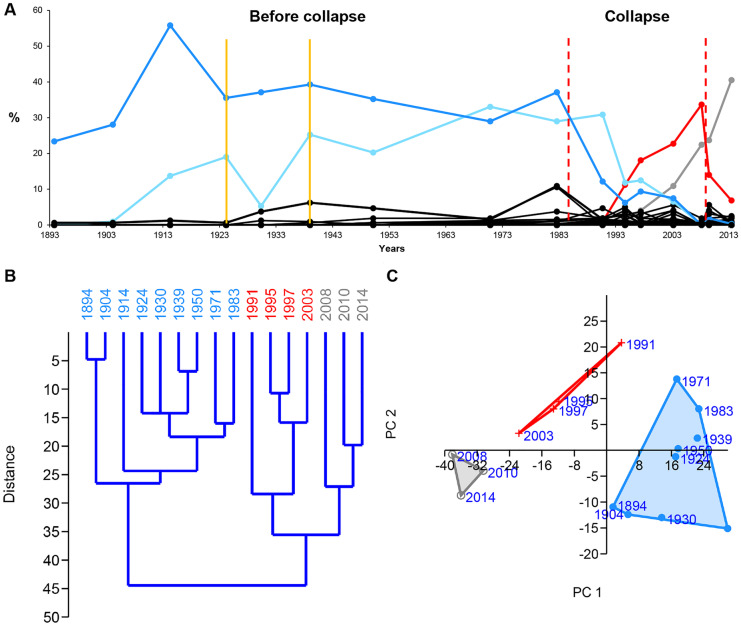
Relative abundance, hierarchical cluster analysis (HCA), and principal component analysis (PCA) of diatoms (Lake Hazen, northern Ellesmere Island, Canada). **A** The relative abundance of diatoms. Blue solid line = pre-collapse dominant species (*Staurosirella pinnata*). Red solid line = collapse dominant species (*Cyclotella comensis*). Light blue solid line = *Staurosira construens*. Grey solid line = recovery dominant species (*Discostella stelligera*). Yellow solid vertical line = environmental step event (summer air temperature rise). Red dashed vertical line = collapse zone boundary. **B** HCA plot and **C** PCA plot. Blue = pre-collapse community, red = collapse community, grey = recovery community. Source of data: Köck et al. (2012) and Lehnherr et al. (2018)

The modern data series shows a recent diatom shift under global warming in Lake Hazen (northern Ellesmere Island, Canada) (Fig. 8). The pre-collapse community represents the period between 1894 and 1983, as the HCA suggests. The collapse zone is between 1991 and 2003. The pre-collapse dominant species is *Staurosirella pinnata* (Lehnherr et al., 2018); the dominant collapse species is *Cyclotella comensis* (Lehnherr et al., 2018). The second most abundant pre-collapse species is *Staurosira construens* (Lehnherr et al., 2018). The ratio of *S. pinnata* and *S. construens* provides information about summer air temperature (Finkelstein & Gajewski, 2008) (see “Identifying environment events” section).

### Identifying environmental events

We had two types of collapse triggering environmental events in the data series: sudden, temporary pulse events and step events that are sudden shifts in an environmental parameter, namely, temperature. We identified the environmental events that probably contribute to the collapses using the literature, PCA, HCA, and the relative abundance of species.

#### KPG

The indicators of the KPG data series clearly show the time of the bolide event. The relative abundance of *P. rudita*, an environmental indicator, has a high peak of 65.70326 Ma (Fig. 3A). At the same time, *N. ripleyensis*, which is the pre-collapse dominant species, drops sharply. In the HCA, the outlier 65.70326 Ma separates the clusters before and after the bolide event (Fig. 3B). The outlier is in the right-hand upper corner in the PCA, away from the clusters (Fig. 3C). Time step 65.70326 Ma is an outlier different from any other time steps in the data series.

#### PE

The Paleocene-Eocene Thermal Maximum occurred at the boundary between the Paleocene and Eocene geological epochs when the temperature rose by 5–8 °C (McInerney & Wing, 2011). The study by Takeda and Kaiho (2007a) indicates the start of this environmental event at 79.925–79.915 mbsf. At this time, *S. brevispinosa*, the dominant species of the pre-collapse community, shoots up. The HCA shows this period as part of cluster 1 (pre-collapse community) but as a separate sub-cluster (Fig. 4B). In the PCA, 79.925–79.915 mbsf are outliers. Based on the relative abundance of species and the HCA (Fig. 4A, B), we identified a previous environmental event, maybe a pulse event that preceded the PETM and may have contributed to the collapse. The HCA shows an outlier in cluster 1 at 79.955 mbsf. After this event, at 79.945 mbsf, the pre-collapse dominant species (*S. brevispinosa*) drops sharply, which is a general sign of environmental events affecting communities dramatically. In PCA, 79.925–79.915 mbsf and 79.955 mbsf are close to each other, suggesting a connection between these time steps (Fig. 4C). We hypothesize that the event at 79.955 mbsf is a pulse event initiating/contributing to the collapse before the PETM.

#### EM1 and EM2

The HCA, the relative abundance of the Late Oligocene-early Miocene nannofossils (Fig. 5A, B), and the relative abundance of the dominant species (Fig. 5A) help identify the environmental events and the direction of the environmental changes. During the Early Miocene (23–16 Ma), the climate started to cool. Sudden, temporary glaciation events occurred between about 21.3 Ma and 20.2 Ma (between approximately 170–180 mbsf and 150–160 mbsf) (Plancq et al., 2013b). The HCA and the PCA show the cooling between clusters 1–3 (Fig. 5B, C). Cluster 4 is different from clusters 1–3. It is probably a warmer period. The relative abundance of the dominant species of community 1–4 indicates the direction of the environmental changes and the environmental events (Fig. 5A). *D. antarcticus* indicates the temporary glaciation events by huge peaks at 180.51 mbsf (community 1) and 169.89 mbsf (community 2), respectively, as well as the glaciation period between 157.45 and 137.21 mbsf (community 3) (Fig. 5A).

EM1 data series covers the beginning of the Early Miocene when the climate started to cool. It includes a temporary glaciation event at 180.51 mbsf (Fig. 6). Based on the experiences with the Modern data series (Fig. 8), we presume a step event and/or a community threshold at 177.92 mbsf (last step before the collapse). At this step, the relative abundance of *Coccolithus* spp. approaches that of *C. floridanus*, while *Dictyococcites* spp., *the dominant species of the collapse zone, starts a sharp increase*. After this step, *C. floridanus* collapses. *Coccolithus* spp. are indicators of interglacial periods (Berggren & Couvering, 1974). The HCA shows this time step as a separate sub-cluster. The step event is hypothetical.

EM2 data series covers a glaciation period in the Early Miocene (Fig. 7). It probably has a temporary warming event (pulse event) at 153.84 mbsf and perhaps at 144.52 mbsf, as well as a possible step event (sudden warming) and/or a community threshold at 137.21 mbsf (the last time step before the collapse), respectively. At 153.84, *D. antarcticus* drops, while *Coccolithus* spp. has a peak (Fig. 7). The same happens at 144.52 mbsf. However, it does not seem to be a significant event at first sight (Fig. 7). Neither PCA nor HCA has an outlier at this time step. Still, the indicators later in the analysis show significant changes in the community structure (Fig. 10). We suggest that even relatively small changes in the abundance of a species can initiate long-term structural changes beyond a critical threshold. The assumed step event and/or community threshold at 137.21 mbsf is similar to the Modern data series at step 1939 (Fig. 8). The steadily increasing *R. minutula* refers to significant warming. The ratio of *R. minutula* and *D. antarcticus* is more than 0.5 at 137.21 mbsf and *Coccolithus* spp. also rises sharply at this point. The increasing relative abundance of *R. minutula* refers to a warming climate (Pujos, 1987). The HCA shows time step 137.21 mbsf as a sub-cluster. The pulse event at 144.52 mbsf and the step event at 137.21 mbsf are hypothetical.

#### Modern

**Fig. 9:**
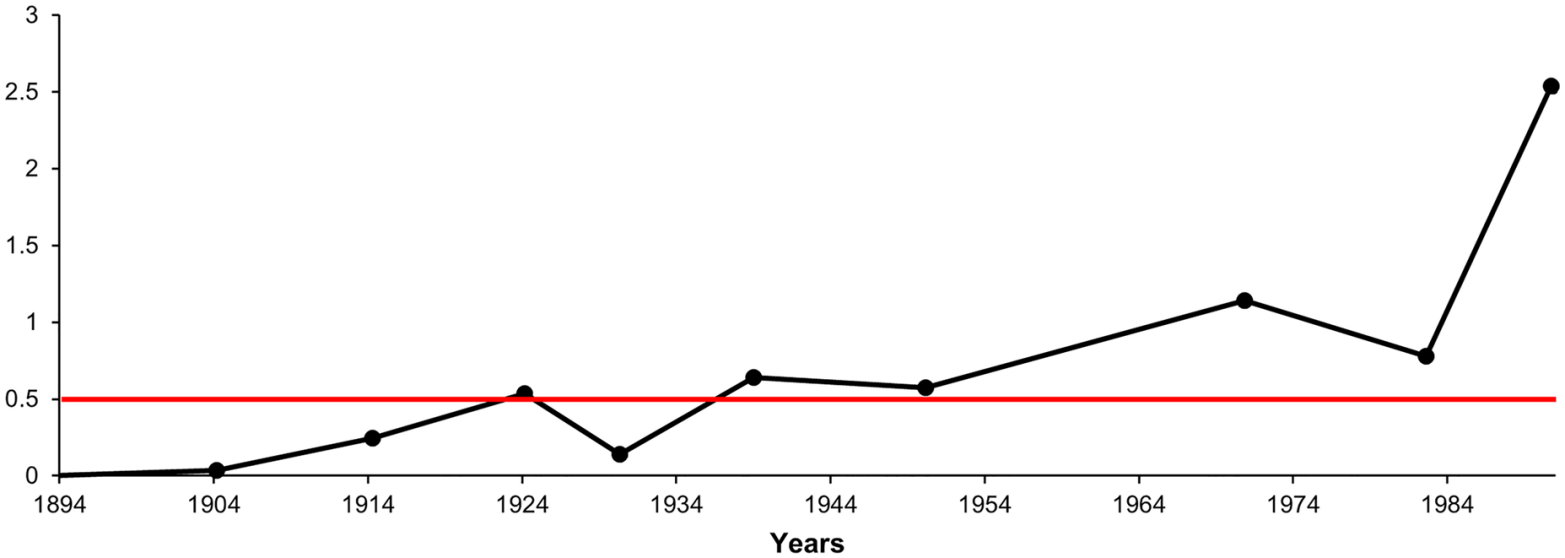
Ratios of *Staurosia construens* and *Staurosirella pinnata*, based on Finkelstein and Gajewski (2008). The increasing ratios refer to warmer summers. Red solid line = critical threshold. The ratio first rose above the threshold in 1924, and it stayed above it after 1939

The main reason for the recent diatom shift in Lake Hazen is the warming climate (Lehnherr et al., 2018). Unfortunately, the study by Lehnherr et al. (2018) does not include information on the local temperature data of the Hazen Lake region for the whole diatom data series. Therefore, we applied an indirect method to be able to detect environmental events. The increasing summer air temperature, the expansion of the growing season, and the earlier melting of the ice sheet (Lehnherr et al., 2018) are probably significant contributors to Arctic melting. The reduced summer albedo due to sea ice and snow cover loss may also be a key component of Arctic amplification (Pithan & Mauritsen, 2014). According to Finkelstein and Gajewski (2008), the lower ratios of *Staurosirella pinnata* to *Staurosira construens* refer to warmer summer air temperatures, while greater ratios suggest cool summers. Using this information, we can learn about the local trends of temperature and perhaps unusual environmental events. To make it more expressive, we use the reverse relationship between these two species, which means that the higher ratios of *Staurosira construens* to *Staurosirella pinnata* refer to warmer summers. In Fig. 9, one can see the ratios of *Staurosira construens* to *Staurosirella pinnata* between 1904 and 1991. The ratio started to increase in 1904 and it shows a general increasing trend. Year 1924 might be an important time step because the ratio of *Staurosira construens* to *Staurosirella pinnata* passes 0.5. The ratio drops temporarily in 1930, then it stays high. After 1983, it shoots up. The local air temperature trends may also reflect regional Arctic trends. The near-surface temperature increased in the Arctic region significantly between the 1920s and 1940s. According to Yamanouchi (2011), Arctic warming from the 1920s to the 1940s is similar to the recent 30–40-year warming.

The probable temporary cooling in 1930 may be a local event (Fig. 9). The figure shows that the ratio has remained high since 1939, and it has been increasing exponentially since 1983, marking the start of the collapse zone. We consider both 1924 and 1939 as important time steps and environmental events in local warming. In 1924, the ratio of *Staurosira construens* to *Staurosirella pinnata* passed 0.5, which is a critical threshold and refers to significantly warmer summers. Year 1939 indicates the onset of the long-term high summer air temperatures.

### Pre-collapse patterns and signals of the indicators

We detected distinct community patterns and signals at the major environmental events and before the collapses and evaluated the performance of the indicators. We examined large-scale signals (long-term trend changes and slope changes) and small-scale signals (short-term peaks and troughs) separately.

We had two types of collapse triggering environmental events in the data series: sudden, temporary pulse events (KPG, PE [presumed], EM1, EM2 [presumed]), and step events that are a sudden stepwise increase temperature (PE, Modern). We also assume a step event and/or a community threshold in EM1 and EM2 data series.

We applied HCA, PCA, and the relative abundance of species to identify the communities, community boundaries, and environmental events. For the detailed description of their signals, see previous parts. Here one can read their short evaluation.

#### HCA

HCA has outliers at the pulse events (except at the second EM2 event) and separate clusters/sub-clusters at the step events (Figs. 3B-5B, 8B). HCA shows communities as separate clusters or sub-clusters. HCA is sensitive to environmental events, and it is an effective tool to assign community boundaries. However, one also needs PCA and the relative abundance of species to interpret the HCA plot.

#### PCA

PCA is not always effective in identifying environmental events, and it does not seem to be a good collapse indicator. However, it helps to detect environmental trends. We applied PCA to visualize communities and to reveal their relationships to each other (Figs. 3C-5C, 8C).

#### Relative abundance of species

The dominant species and the environmental indicators are particularly vulnerable to environmental changes. Hence, the relative abundance of species is a helpful indicator in identifying the environmental events. The decline of the dominant species and, at the same time, the increase of a subdominant species/environmental indicator/opportunist species usually refer to some significant environmental changes (Figs. 3A-5A, 6-7, 8A). Temporary changes imply a not (immediately) fatal pulse event; the long-term decline of the dominant species is related to step events/community thresholds or a fatal pulse event. When the ratio of the second most abundant species and the declining dominant species becomes less than 0.5, it is a critical time step referring to a step event/community threshold (Figs. 8A, 9). When the dominant species is an opportunist (see PE data series) (Fig. 4A), it can take advantage of the environmental deterioration in the short run, which delays its decline. We use it along with the HCA to detect the community boundaries. When the dominant species changes in a sub-cluster, it indicates a community shift/extinction.

#### Total abundance

**Fig. 10:**
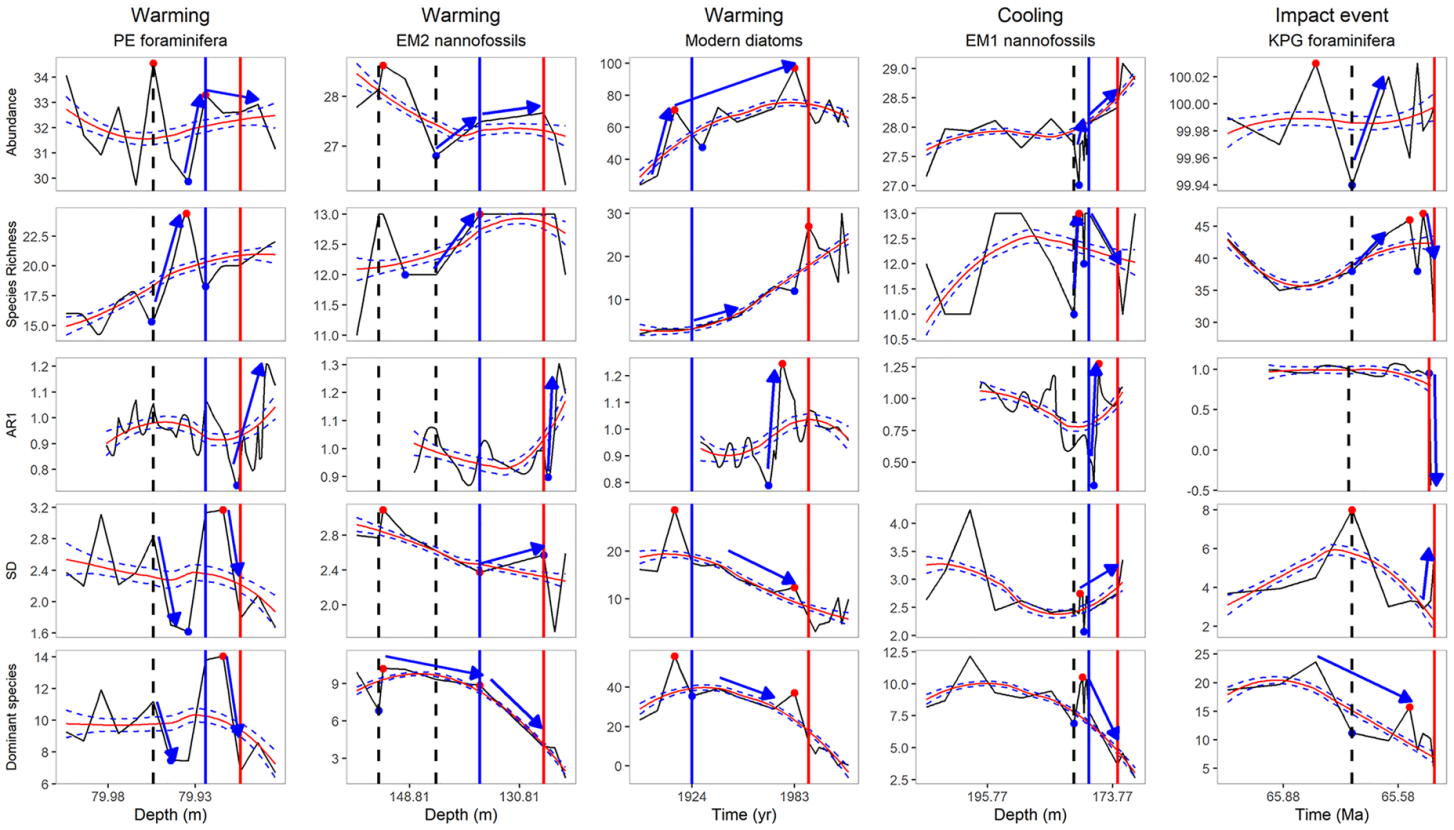
Small-scale signals and large-scale signals before collapses. Black dashed vertical line = pulse event (EM2 second event is hypothetical). Blue solid vertical line = step event (EM2 and EM1 events are presumed). Red solid vertical line = collapse zone boundary. Solid dots = small-scale signal (red = peak, blue = trough). Blue arrow = large-scale signal. Red curve with blue dashed curves = 95% pointwise confidence band

We detected a general pattern of the total abundance before collapses in all of the data series. The abundance first increases sharply. Then it starts a segment with a significantly smaller slope lasting approximately until the collapse zone boundary (Fig. 10). The sharply increasing segment starts at a local or absolute minimum: in the case of EM1, KPG, and EM2 data series, at a pulse event/an assumed pulse event, in the case of PE data series, after the assumed pulse event. We presume that the total abundance drops under a critical threshold at this point resulting in a minimum. No obvious minimum point is visible in the total abundance of the Modern data series because this point might be before 1894 (the beginning of the Modern time series). The slope of the total abundance begins decreasing considerably at the step events/community thresholds of PE, EM2, and EM1 data series.

The figures of the relative abundance of species (Figs. 3A, 5A, and 8A) show that at the minimum of the total abundance, the relative abundance of the dominant species also drops. In contrast, subdominant species, environmental indicators, opportunistic species shoot up, except in the PE data series (Fig. 4A). We think that the pulse events push the dominant species into the stress zones of their tolerance ranges, in most cases causing a minimum of the total abundance. PE data series is different because the dominant species (*S. brevispinosa*) is an opportunist (Giusberti et al., 2016). Hence, it outcompetes other species even under stress. The sudden increase of the total abundance after the minimum is a countertrend. During this countertrend, the dominant species already shows a decreasing trend, except in the case of PE data series again (Fig. 4A). In the PE data series, the sudden increase of the dominant species ensures the countertrend of the total abundance.

In the segment where the total abundance has a small slope (after the step event), the significant increase of other species counterbalances the decrease of the dominant species. In this segment, the relative abundance of the dominant species is much lower than it was in the previous period. In the PE data series, the decline of the dominant species is delayed.

The small-scale signs of the total abundance (both peaks and troughs) (Fig. 10) reflect the changes in the relative abundance of the dominant species, the environmental indicators, and the opportunistic species (Figs. 3A, 4A, 5A, and 8A). They are related to events, trend changes, and boundaries. Trend changes or a significant change of the slope can follow great small-scale signs. It means that the environmental event is so overwhelming that it has a long-term effect on the community and causes the decline of the dominant species. In the PE and Modern data series, we find a peak near the step events. This small-scale sign is followed by a trend change or the slope change introducing a segment with a gentle slope. We suggest that this segment is the consequence of passing a critical threshold, for example, a sudden, stepwise long-term increase of the temperature forcing the pre-collapse community in the stress zone of their tolerance range. In the case of the EM1 and EM2 data series, we are not sure whether a step event (abiotic cause) or a community threshold (biotic cause) initiates the segment with a small slope.

##### Evaluation

The total abundance has a uniform pre-collapse pattern with distinct and explicable segments giving general information about the condition of the community before the collapse. The dominant species probably has a vital role in shaping the pattern of the total abundance of the community before the collapse. It gives small-scale and large-scale signs at environmental events. It typically provides sharp, small-scale signs (troughs or peaks) at the pulse events and large-scale signs (steep increase) after the pulse events, while the step events/community thresholds initiate large-scale signs, relatively longer segments with a significantly smaller slope. However, it may not be a good indicator for the collapse zone boundary. At the collapse zone boundary, it gives different and sometimes late signals (upward or downward trend), though it starts a decreasing trend uniformly in the warming data series (PE, EM2, Modern). The total abundance of the KPG is quite different, strongly fluctuating, and probably does not have a critical-slowing-down segment.

##### Notes

The KPG data series includes few data. Because of the interpolation, the total abundance seems to give a signal before the event. However, the signal triggered by the probable impact event is the absolute minimum. The total abundance of the KPG data series is strongly fluctuating, making the analysis more difficult. Despite this fact, we did not smooth the data to preserve the small-scale signs or avoid their displacement. Smoothing can affect even the direction of large-scale signs.

#### Species richness

Pulse events can initiate a sharper increasing trend after a sudden trend change or an increase of the slope, see PE presumed pulse event, EM2 second pulse event (presumed), EM1, and KPG pulse events (Fig. 10). A small-scale sign (minimum) does not always indicate the beginning of the steeper increasing trend. However, at the end of the upward trend, peaks are typical. We propose that the decline of the dominant species and the rise of other species cause this pattern. The sudden drop of the relative abundance of the dominant species and the shoot-up of other species evoke the peaks at the end of the upward trend. At the step events or community thresholds, we did not detect a robust increase. Moreover, we observed a decreasing trend on one occasion.

After the step event, the species richness of the PE pre-collapse community continues its general increasing trend, the species richness of the EM 2 data series stagnates at a high level, and that of the Modern data series increases slightly. After the presumed EM1 step event or community threshold, the species richness starts increasing before the collapse.

##### Evaluation

The sudden increase of the species richness is frequent after pulse events. However, step events or community thresholds do not always provoke significant changes, and these changes do not always point in one direction. Small-scale signs do not always indicate the events (for instance, Modern and KPG events). Another observation is that the maximum at the end of the increasing trend sometimes locates before the collapse zone boundary, sometimes right at the boundary. In EM1 and KPG data series, species richness starts decreasing before the collapse zone boundary. Species richness may not be a good indicator for the approaching collapse zone because of the diversity of the signals. However, applying it all together with the abundance, they can give helpful information about the general condition of the pre-collapse community.

#### Standard deviation

The standard deviation does not have a uniform pattern before the collapses. It has a decreasing trend before PE and Modern collapses (warming events), while it increases in the case of EM2 (warming), EM1 (cooling), and KPG (impact event) communities before the collapse zone boundary. Near the events, the change points and the slope change of the standard deviation provide small-scale signs (peaks and troughs).

##### Evaluation

It usually has small-scale signs at the environmental events (peaks and troughs). Since this statistical indicator does not have any consistent signs, we do not consider it reliable.

##### Notes

We applied blank cells where we had no data, and we did not apply a rolling window. Authors usually use standard deviation with a rolling window in articles on critical slowing down (Wouters et al., 2015). According to Tu et al. (2020), standard deviation (with a rolling window) is a better collapse indicator than AR1.

#### AR1

According to Dakos et al. (2015), critical slowing down causes a rise in temporal correlation. In PE, EM2, Modern, and EM1 data series (warming and cooling events), AR1 started a sharp increase from a minimum point before or at the collapse zone boundary. However, AR1 does not increase, only fluctuates before the KPG collapse (impact event) and drops at the collapse zone boundary (Fig. 10). KPG is a transient collapse as a consequence of a random asteroid hit. Additionally, the KPG data fluctuate enormously before and after the event. All of these might decrease the effectiveness of the AR1 indicator.

Dakos et al. (2008) suggest that abrupt climate change is related to critical slowing down and tipping point. It might be an explanation for the increase of AR1 in other data series. AR1 starts to increase before the collapse zone boundary in the Modern and EM1 data series. However, it shoots up only at the beginning of the collapse zone in the PE and EM2 data series.

##### Evaluation

AR1 is not a good environmental indicator. It indicates or forecasts the collapse zone in case of warming and cooling events. Sometimes it might be late. It shoots up before/near the collapse zone boundary. However, it does not provide a universal signal. It gives a decreasing large-scale sign at the KPG collapse zone boundary.

#### Relative abundance of dominant species

The declines of the dominant species have both similarities and differences in the observed collapses. PE includes two events affecting the dominant species. After the presumed pulse event, the relative abundance of the dominant species drops to a local minimum, but it does not cause a total collapse. The final decline of the dominant species begins due to PETM. The dominant species starts a long-term decrease leading to a collapse after the first pulse event of EM2, the Modern step event, and the KPG bolide event. The EM1 dominant species shows a long-term decreasing trend, but its decline and collapse accelerate after the temporary glaciation. The EM1 pre-collapse period is likely to be a long-term cooling affecting the dominant species negatively, and the transient, glacial event acts as an accelerator of the collapse. During warming and cooling events, the decline of the dominant species starts slowly (distinctive segment with a small slope), but then it speeds up approaching the collapse zone boundary. The KPG bolide event causes the fall of the dominant species right immediately. At the collapse zone boundary, it does not change a trend.

##### Evaluation

The dominant species are sensitive to major environmental events. The relative abundance of the dominant species has a decreasing trend before all the analyzed collapses result from some significant environmental event. Long-term trend changes or the changes of the slope and then the final decline follow these events. At the trend changes and the changes of the slope, we can see peaks as small-scale signs. Troughs as a small-scale sign are typical at the pulse events. It does not give a signal at the collapse zone boundary.

##### Notes

The KPG data series consists of relatively little data that can lead to misinterpretation. The interpolation of the data might imply that the relative abundance of the dominant species has a warning sign before the event. However, the sign is the local minimum at the bolide event. The dominant species of the PE community shows a bit different pattern from the dominant species of other data series. At the beginning of the PETM, the relative abundance of the dominant species has an increasing trend with a small slope. It starts a sharp decrease later. We explained this lagged decrease because the dominant species, *S. brevispinosa*, is probably an opportunist species capable of adapting to changing environments (Giusberti et al., 2016).

## Discussion and conclusions

Our results show that AR1 is not a good environmental event indicator in general. PCA is not a reliable environmental event indicator, either. The rest of the indicators are more sensitive to the pulse events than to the step events.

The abundance, the relative abundance of species, and the relative abundance of dominant species give small-scale signs at *major pulse events*. (The dominant species gives only a weak signal at the second presumed EM2 pulse event.) They often give large-scale signs, but not in every case. The species richness gives a large-scale sign (increase) and sometimes a small-scale sign at the pulse events. The abundance and the species richness often start a sharp increase (large-scale sign) at pulse events. The standard deviation gives a small-scale sign (peaks) at the pulse events, but its large-scale signs are inconsistent (decrease or increase). The HCA has an outlier at the pulse events except at the second presumed EM2 pulse event. PCA not always has an outlier at the pulse events.

Fewer indicators give consistent, reliable signs at the *step events/community thresholds* before the collapses. The relative abundance of the dominant species gives both small-scale signs (peaks) and large-scale signs (decreasing trend) at the step events/community thresholds. The relative abundance of the species is also sensitive to step events. However, to interpret the results, one needs to have background information about the dominant species and the environmental indicators. The abundance has a trend change or the change of the slope at step events where the slope of the trend line decreases dramatically. (The modern data series is too short to see exactly where the segment with a gentle slope starts.) The HCA mainly shows step events as separate sub-clusters.

Some indicators sensitive to environmental events (abundance, species richness, relative abundance of dominant species) are not reliable *indicators of the collapse zone*. Applying the HCA, the PCA, and the relative abundance of species together effectively identify collapse zone boundaries. AR1 is a good collapse zone indicator in climate change data series (PE, EM1–2, Modern). In EM1 and Modern data series, it gives an earlier signal (an increasing trend) than HCA, PCA, and the relative abundance of species together. However, during the KPG bolide event, it fluctuates before the collapse and drops sharply at the collapse zone boundary. Standard deviation without a moving window is not a good collapse indicator. Probably, using a moving window can enhance its performance.

*Unusual environmental events* are important causes of community collapses (Johansson et al., 2020; Román-Palacios & Wiens, 2020). Both pulse and step events can trigger a collapse. We believe that indicators sensitive to environmental events like species richness, abundance, the relative abundance of the dominant species, HCA, and the relative abundance of species can give important information about the deteriorating processes in the community long before the collapses.

Based on the analyzed data series, we assume that a *step event* (abiotic cause of the collapse) might precede the collapses triggered by climate change where the community gets beyond a critical threshold (biotic cause of the collapse). It might cause the long-term decline of the dominant species and the rise of other species. These processes develop the distinctive pattern of some indicators (the small slope of the total abundance, the decreasing trend of the dominant species, the increasing ratio of some environmental indicators and the dominant species). If our hypothesis is confirmed, we should focus on detecting pre-collapse step events in the data series because they give earlier signals than the collapse zones.

During our analysis, we distinguished between small-scale signals (peaks and troughs) and large-scale signals (long-term trend changes and sudden, long-term changes of the slope). Small-scale signs (maximum-minimum points) can precede large-scale signs. Hence, they can indicate the forthcoming trend changes. When several indicators simultaneously give a significant small-scale sign, they refer to important events (major environmental events, collapse zone boundaries). The frequently used critical slowing down indicators do not focus on small-scale signs. We propose that it might be helpful to include small-scale signals in collapse-related studies as they often precede large-scale signals.

We believe it is essential to forecast the collapse zones and recognize the antecedent collapse-triggering, unusual environmental events. We suggest that the authors apply several statistical indicators to reveal pre-collapse community patterns and signals since some indicators are sensitive to pulse events, others to step events, and some indicators detect the best collapse zones. Another reason for using several indicators is that it is easier to track less obvious yet significant environmental events striking communities with decreased resilience.

A future task is to test more statistical indicators to see whether they are sensitive to pulse events, step events, or collapse zones. For example, constancy, dominance, Shannon-H, diversity indices, or indicators combined with AR1 might reveal some new aspects of pre-collapse communities.

## Data Availability

The data that support the findings of this study are all available from the corresponding author upon request.
